# Twisted Anthracene-Fused BODIPY: Intersystem Crossing and Torsion-Induced Non-Radiative Relaxation of the Singlet Excited State

**DOI:** 10.3390/molecules31030524

**Published:** 2026-02-02

**Authors:** Andrey A. Sukhanov, Yanran Wu, Yuqi Hou, Bei Li, Yu Dong, Jianzhang Zhao, Violeta K. Voronkova, Bernhard Dick

**Affiliations:** 1 Zavoisky Physical-Technical Institute, FRC Kazan Scientific Center of Russian Academy of Sciences, Kazan 420029, Russia; 2 State Key Laboratory of Fine Chemicals, School of Chemical Engineering, Dalian University of Technology, E-208 West Campus, 2 Ling Gong Road, Dalian 116024, China; yanranwu@mail.dlut.edu.cn (Y.W.);; 3 School of Chemical Engineering, Ocean and Life Sciences, Dalian University of Technology, Panjin 124221, China; 4 Lehrstuhl für Physikalische Chemie, Institut für Physikalische und Theoretische Chemie, Universität Regensburg, Universitätsstr. 31, D-93053 Regensburg, Germany

**Keywords:** boron–dipyrromethene, intersystem crossing, spin–orbit coupling, triplet state, conical intersection

## Abstract

The photophysical properties of a BODIPY derivative with the highly twisted molecular structure of anthracene-fused boron–dipyrromethene (**AN-BDP**) were studied with steady-state and time-resolved spectroscopic methods. The fused anthryl and the BDP units in **AN-BDP** units both adopt distorted geometry (with ca. 10° of torsion), and there is large dihedral angle between the two units (ca. 49.7°). Interestingly, the fluorescence quantum yields are highly dependent on the solvent polarity (59~3%, from toluene to acetonitrile), yet the fluorescence emission wavelength does not change in different solvents. Nanosecond transient absorption spectra indicate that the triplet state is long-lived, with an intrinsic triplet state lifetime of 551 μs. Interestingly the severely twisted structure only shows a moderate intersystem crossing (ISC) yield (10%). Femtosecond transient absorption spectra indicate slow ISC (>1.5 ns), which is in agreement with the fluorescence lifetime (2.3 ns). Time-resolved electron paramagnetic resonance (TREPR) spectra show smaller zero-field-splitting *D* and *E* tensors as (−71.4 mT, 16.7 mT, respectively) compared to the triplet state of the iodinated native BDP (*D* = −104.6 mT, *E* = 22.8 mT), inferring that the triplet-state wave function of the new compound is delocalized over the twisted molecular framework. The theoretical computation indicated a solvent-polarity-dependent energy barrier for the relaxed S_1_ state to a conical interaction (CI) of the S_1_ and the S_0_ state potential curves, which agrees with the weaker fluorescence in polar solvents.

## 1. Introduction

Triplet photosensitizers (PSs) have attracted much attention in recent years, due to the significance of this kind of novel organic molecules in fundamental study of intersystem crossing (ISC) [1,2,3,4,5], as well as their critical roles in applications such as photocatalysis [6,7,8,9], photodynamic therapy (PDT) [10,11,12,13,14], and triplet–triplet–annihilation photon up-conversion [15,16,17,18,19,20,21]. The ISC ability of triplet PSs is one of the most important properties of these molecules. However, for most of the planar aromatic hydrocarbons, ISC is strongly forbidden because of the large electron exchange energy (*J*), leading to the large S_1_/T_1_ states energy gap, and the weak spin–orbit coupling [22]. Typical methods to enhance the ISC in organic chromophores include using the heavy atom effect (incorporate Pt, Ir, Ru, Br and I atoms in chromophore) [12,13,23,24,25,26], the exciton coupling effect [27,28], establishing n-π *↔ π-π * transitions (El Sayed’s rule for ISC) [29], radical enhanced ISC [30], and charge-recombination-induced ISC [31]. Some drawbacks exist for these conventional methods: for instance, the high cost of precious metal ions, shortened triplet state lifetimes as a result of the heavy atom effect or the radical enhanced ISC, and the difficulties in synthesis [32]. Thus, it is necessary to develop new approaches, based on a simple molecular structure motif, to enhance the ISC in organic chromophores and solve the above problems, which is important for both fundamental photochemistry and for the various applications of these findings.

**Figure 1:**
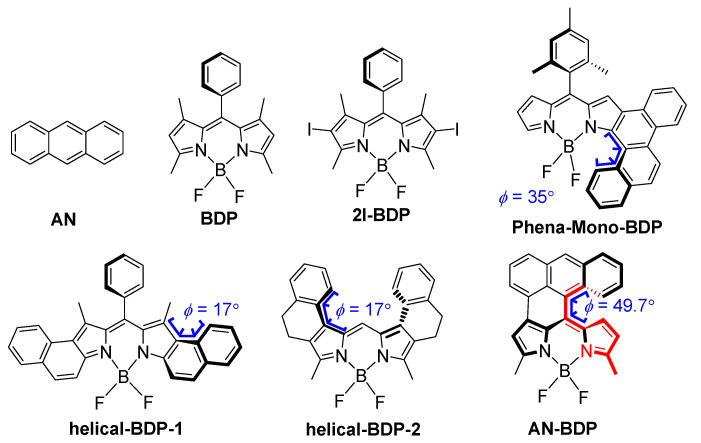
Molecular structures of the compound **AN-BDP**, and the recently reported twisted BODIPY derivatives showing ISC (**Phena-Mono-BDP**, **helical-BDP-1** and **helical-BDP-2**) and reference compounds (**AN**, **BDP** and **2I-BDP**). The colored part of **AN-BDP** shows the torsion of the molecule.

Concerning this aspect, the twisted π-conjugated framework of a molecular-structure-induced ISC is in particular of interest. Some compounds with twisted molecular structures are known to show efficient ISC: for instance, fullerenes C_60_/C_70_ [33] and helicenes [34,35]. However, these compounds alone are not ideal triplet PSs because of their weak absorption of visible light, poor solubility, and challenging derivatization chemistry. Recently, a few other chromophores with twisted geometry showing ISC have been reported: for instance, perylene bisimide (PBI) [36,37,38,39]. Recently, we and Hasobe et al. found that twisted boron–dipyrromethene (BODIPY) also show efficient ISC (Figure 1) [40,41,42]. However, our understanding of the structure–ISC efficiency relationship of these compounds (especially the BODIPY compounds) is far from mature. For instance, we found that the slightly twisted BODIPY derivative show efficient ISC (torsion angle: 7.5°, triplet state quantum yield: 52%) [40], yet some BODIPY derivatives having severely twisted molecular structure show less efficient ISC (torsion angle: ca. 17°, triplet state quantum yield: 16%) [41]. In another twisted BODIPY (**helical-BDP-2**) with similar torsion angle (ca. 17°), the triplet state quantum yield is as high as 56% [42]. This trend is different from the helicene compounds, for which the more twisted molecular structure usually induces more efficient ISC [34]. Therefore, it is necessary to attain more in-depth understanding of the effect of twisting of the molecular structure on the ISC efficiency with more examples.

Another interesting aspect of the ISC of the twisted BODIPY is the electron spin selectivity, or the population rates of the three sublevels (T_x_, T_y_, T_z_) of the T_1_ state of the twisted BODIPY derivatives; this is one of the fundamental photophysics principles of ISC [43,44,45,46]. However, it is not possible to study this property with normal optical spectral methods. Recently, we studied the triplet state of twisted BODIPY derivatives and their electron spin polarization (ESP) with time-resolved electron paramagnetic resonance (TREPR) spectroscopy, and we found that the electron spin selectivity of the ISC of the twisted BODIPY chromophore is highly dependent on the molecular structure [40,42]. However, more examples are needed to fully unveil the electron spin selectivity of the ISC of the compounds with a twisted molecular structure.

In order to study the relationship of twisting ISC efficiency, herein, we studied an anthracene-fused BODIPY derivative (**AN-BDP**, Figure 1) [47]. Both the partially fused anthryl moiety and the BODIPY moiety are highly distorted, and the torsion between the two moieties is large (49.7°, see later sections for detail). The photophysical properties of the compound were studied with steady-state and time-resolved spectroscopic methods; ISC and long-lived triplet state were observed. The TREPR spectra show the electron spin selectivity of the ISC in the twisted BODIPY derivative.

## 2. Results and Discussion

### 2.1. Molecular Structure of the Twisted BODIPY

Native BODIPY has a planar molecular structure, which makes the electron exchange energy (*J*) large; thus, the large S_1_/T_1_ energy gap inhibits ISC. Inspired by the twisted π conjugation-framework-induced ISC in helicene [34,35], PBI [36,38,39], and the recently reported twisted BODIPY derivatives (**helical-BDP-1** [40], **helical-BDP-2** [42] and **Phena-Mono-BDP** (*Φ*_Δ_ = 63% in DCM) [41]), herein, we studied another BODIPY derivative which has highly twisted molecular structure, **AN-BDP** [47]. This compound was reported previously, but no detailed studies on its geometry or ISC were presented [47]. The difference between **AN-BDP** and **Phena-Mono-BDP** is that the BODIPY core in the former is severely distorted (see later section); it is almost planar for the latter [41]. This different geometry for the BODIPY core may have significant impacts for the photophysical properties: for instance, the non-radiative decay of the S_1_ state to the ground state [48]. Moreover, the fluorescence quantum yield and the ISC quantum yield may be also affected.

### 2.2. UV–Vis Absorption and Fluorescence Spectra

**Figure 2:**
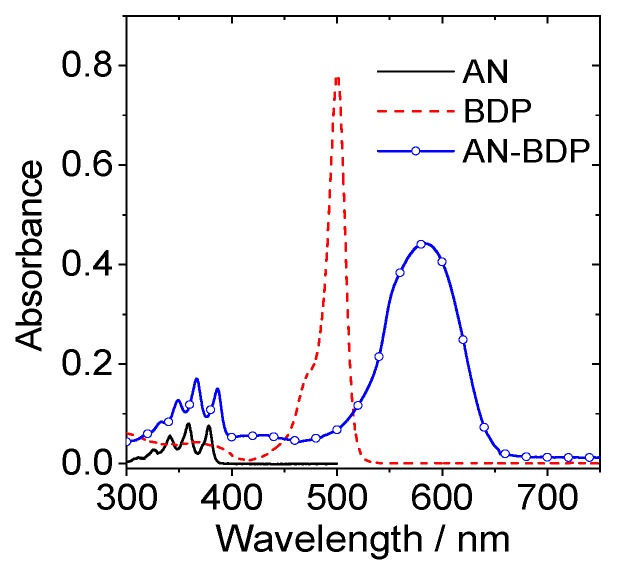
UV–vis absorption spectra of **AN**, **BDP** and **AN-BDP** in DCM, *c* = 1.0 × 10^−5^ M, 20 °C.

The UV–vis absorption band of **AN-BDP** is centered at 583 nm, which is broad and structureless (Figure 2). These features are different from the absorption of native BODIPY [49,50,51], as well as the compact dyad with anthryl moiety attached at the *meso*-position of BODIPY [52,53]. These results indicate that π-conjugation framework of **AN-BDP** is different from the native BODIPY. The UV–vis absorption of **AN-BDP** is also different from the fully fused anthryl-BDP analogues (which is with planar π-conjugation framework), which show red and near IR absorption bands centered at 606 nm (*ε* = 7200 M^−1^ cm^−1^), 650 nm (*ε* = 11,300 M^−1^ cm^−1^), 760 nm (*ε* = 5600 M^−1^ cm^−1^), and 826 nm (*ε* = 5700 M^−1^ cm^−1^) [54].

**Figure 3:**
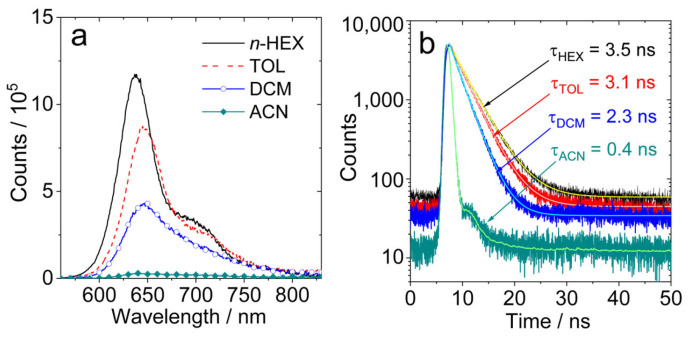
(**a**) Fluorescence spectra of **AN-BDP** in different solvents. Optically matched solutions were used, *λ*_ex_ = 550 nm, *A*_550nm_ = 0.22; (**b**) fluorescence decay traces of **AN-BDP** in different solvents. *c* = 1.0 × 10^−5^ M, *λ*_ex_ = 510 nm, 20 °C.

The fluorescence band of **AN-BDP** is centered at 645 nm, which is red-shifted more when compared with the fluorescence of native BODIPY (ca. 500 nm) [55]. Interestingly, the fluorescence intensity is reduced in polar solvents, but the fluorescence emission wavelength is not red-shifted (Figure 3). This is unusual, and it indicates that the quenching of the fluorescence in polar solvent is not due to the energy gap law. We propose that there is a non-radiative relaxation channel for **AN-BDP**, which is more efficient in polar solvents. This postulate is confirmed by the theoretical studies (see later section, the theoretical calculation shows that the energy barrier for non-radiative decay of the S_1_ state becomes smaller in polar solvents). The fluorescence behavior of **AN-BDP** is also different from the previously reported anthryl-BODIPY dyads, for which charge transfer (CT) emission band was observed (ca. 650 nm), as well as the LE emission (ca. 515 nm) [47,52,53].

The fluorescence lifetimes of **AN-BDP** in different solvents were studied with the time-correlated single-photon counting (TCSPC) technique (Figure 3b). From non-polar solvents to polar solvents, the fluorescence lifetimes are shortened gradually. For instance, the fluorescence lifetime in HEX is 3.5 ns, whereas it is 3.1 ns, 2.3 ns and 0.4 ns in TOL, DCM and ACN, respectively. This result indicates that a non-radiative decay channel is more efficient in polar solvents. Based on the fluorescence quantum yields and the fluorescence lifetimes (Table 1), the radiative decay rate constants (*k*_r_) and the non-radiative decay rate constants (*k*_nr_) in different solvents were calculated; the results show that *k*_nr_ is strongly influenced by the solvent. For **helical-BDP-2**, the fluorescence quantum yields are solvent-independent, and the fluorescence lifetimes are solvent-polarity-independent (3.6~4.7 ns) [42]. For the **Phena-Mono-BDP**, the fluorescence quantum yields are almost solvent-polarity-independent (13~22% in most solvent, 4% in DCM), and the fluorescence lifetimes are in the range of 2.48~3.53 ns [41]. Thus, the solvent-polarity-dependent fluorescence quantum yield and lifetimes of **AN-BDP** are unique in these twisted BODIPY derivatives. Moreover, we noted that the non-radiative relaxation of the S_1_ state of **AN-BDP** to the ground state is significant (i.e., the sum of the fluorescence quantum yield and the ISC quantum yield of **AN-BDP** in a specific solvent is much less than unity), which is different from **Phena-Mono-BDP** [41], **helical-BDP-1** and **helical-BDP-2** [40,41,42]. The efficient non-radiative decay of the S_1_ state of **AN-BDP** may be due to the puckered geometry of the BODIPY core [48,56], which is different from the planar BODIPY core in **Phena-Mono-BDP** [41]; for both the **helical-BDP-1** and **helical-BDP-2**, the BODIPY core adopts an almost planar geometry [40,42].

### 2.3. Electrochemistry Study

**Figure 4:**
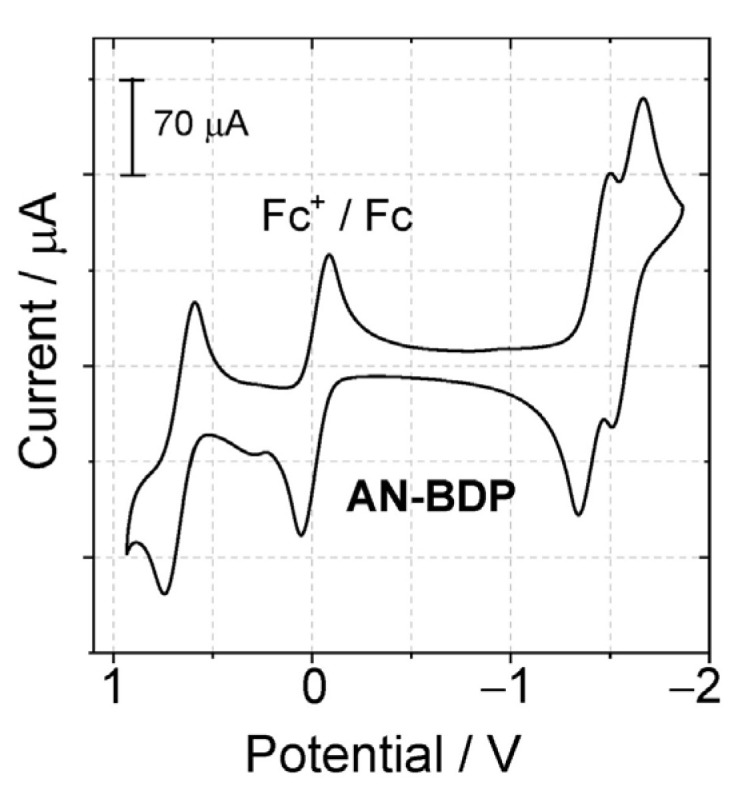
Cyclic voltammograms determined for **AN-BDP** in deaerated DCM containing 0.10 M Bu_4_N[PF_6_] as supporting electrolyte and with Ag/AgNO_3_ as reference electrode. Scan rate: 100 mV/s. Ferrocene (Fc) was used as internal reference (set as 0 V in the cyclic voltammograms), *c* = 1.0 × 10^−3^ M, 25 °C.

The redox potentials of the dyads were measured with cyclic voltammetry (Figure 4). For **AN-BDP**, a reversible oxidation wave was observed at +0.65 V (vs. Fc/Fc^+^), two reversible reduction waves, at −1.40 V and −1.61 V (vs. Fc/Fc^+^) were observed. According to the molecular orbital analysis, the HOMO is mainly confined on the BODIPY part, and the LUMO is mainly confined to the anthryl part. However, it should be noted that the separation of the HOMO and LUMO is not distinct, i.e., the π-conjugation between the BODIPY part and the anthryl part is significant. The HOMO and LUMO energy levels are influenced by the torsion of the π-conjugation framework. This effect may further lead to the change of the energy levels of the S_1_ state and the T_n_ states, as well as their energy matching, which eventually may lead to variation of the ISC ability of the chromophores having a twisted π-conjugation framework.

### 2.4. Nanosecond Transient Absorption (ns-TA) Spectra

**Figure 5:**
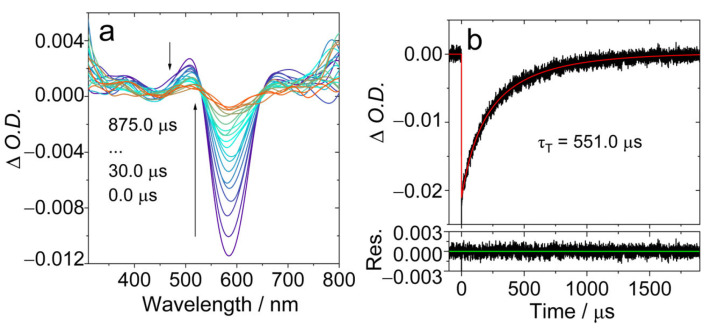
Nanosecond transient absorption spectra of **AN-BDP** in deaerated DCM. (**a**) Transient absorption spectra and (**b**) decay trace at 585 nm. *λ*_ex_ = 580 nm, *c* = 3.0 × 10^−5^ M, 20 °C. The *τ*_T_ is the intrinsic lifetime obtained by fitting *τ*_T_ in two different concentrations. The arrows in (**a**) indicate the evolution of the spectrum along with increasing of the delay time after laser flash.

The ns-TA spectra of **AN-BDP** were studied (Figure 5). Upon pulsed laser excitation, a ground-state bleaching (GSB) band centered at 586 nm was observed, together with the weak excited state absorption (ESA) bands centered at 506 nm and 680 nm, respectively (Figure 5a). This profile may be due to the superposition of the GSB and the ESA bands, i.e., there is probably a broad ESA band in the range of 450 nm–700 nm. The ESA bands are similar to that of the native BDP [26]. The intrinsic triplet state lifetime was determined as 551.0 μs, based on a kinetics model with the TTA self-quenching effect considered in [53,55]. This lifetime is much longer than that of the triplet state of the native BDP (ca. 82 μs) [53] and **helical-BDP-2** (197.5 μs) [57], but it is comparable to the recently reported twisted BODIPY (**helical-BDP-1**, 492 μs) [40].

**Figure 6:**
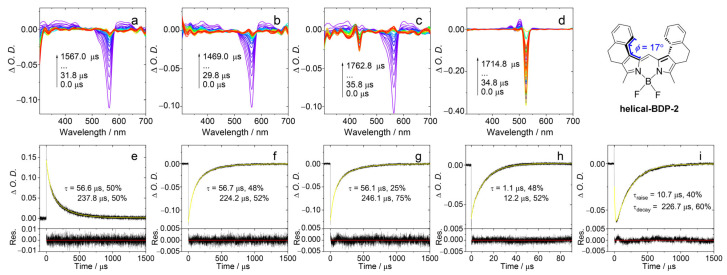
Nanosecond transient absorption spectra and decay traces in deaerated DCM. (**a**) Spectrum of **helical-BDP-2** alone. (**b**–**d**) Spectra of **helical-BDP-2** (triplet donor) in the presence of the triplet acceptors 9,10-Diphenylanthracene, Perylene, and PBI, respectively. (**e**–**h**) Corresponding decay traces monitored at 560 nm for conditions (**a**) to (**d**), respectively. (**i**) Decay trace at 505 nm for **helical-BDP-2** with **PBI**. *λ*_ex_ = 550 nm, *c*[donor] = 5.0 × 10^−6^ M, *c*[acceptor] = 2.0 × 10^−5^ M, 20 °C. The arrows in (**a**–**d**) indicate the evolution of the spectrum along with increasing of the delay time after laser flash.

The T_1_ state energy of **AN-BDP** was calculated as 1.02 eV with time-dependent density functional theory (TDDFT) method. In comparison, the T_1_ state energy of **Phena-Mono-BDP** was determined as 1.42 eV (phosphorescence method) and the S_1_ state energy is 1.99 eV [41]. The T_1_ state energy of **helical-BDP-2** is 1.60 eV (TDDFT computation) [42], which is in good agreement with the triplet–triplet energy transfer (TTET) experimental results (1.2–1.7 eV, Figure 6).

We used TTET experiment to estimate the T_1_ state energy level of **helical-BDP-2**. In this experiment, **helical-BDP-2** is used as the energy donor, while three different compounds (9,10-Diphenylanthracene, perylene, and PBI) were used as energy acceptors. After selectively exciting **helical-BDP-2** at 550 nm, we measured the quenching of its triplet state lifetime to determine the range of its T_1_ energy, as shown in Figure 6. The T_1_ state energies of the acceptors were calculated as follows: 9,10-Diphenylanthracene, 1.77 eV; perylene, 1.53 eV; and PBI, 1.24 eV. After selectively exciting **helical-BDP-2** at 550 nm, the ns-TA spectrum showed a GSB band centered at 560 nm (Figure 6a). When 9,10-Diphenylanthracene was introduced, the decay lifetime at 560 nm (Figure 6f) remained essentially the same as without any acceptor (Figure 6e). No characteristic triplet state absorption of 9,10-diphenylanthracene was detected. This indicates that the T_1_ state energy level of 9,10-Diphenylanthracene is much higher than that of **helical-BDP-2**. Note upward energy transfer is thermodynamically forbidden. Upon addition of perylene, a GSB signal of perylene at 435 nm can be observed in the spectrum (Figure 6c). However, the decay lifetime monitored at 560 nm shows almost no change (Figure 6g). This suggests that the T_1_ state energy levels of **helical-BDP-2** and perylene are relatively close. Furthermore, the presence of PBI caused the GSB band centered at 560 nm to disappear rapidly, while a new GSB band centered at 525 nm emerged, attributed to the GSB band of PBI (Figure 6d). This new signal exhibits a distinct rise-and-decay biphasic feature, corresponding to the generation and subsequent decay of the PBI triplet state, confirming the occurrence of highly efficient energy transfer. Since the T_1_ state energy level of PBI (1.24 eV) is lower than that of **helical-BDP-2**, while 9,10-diphenylanthracene caused no effective quenching, the experimental results indicate that the T_1_ state energy level of **helical-BDP-2** falls within the range of 1.24–1.77 eV, likely around 1.5 eV.

### 2.5. Femtosecond Transient Absorption (fs-TA) Spectra

**Figure 7:**
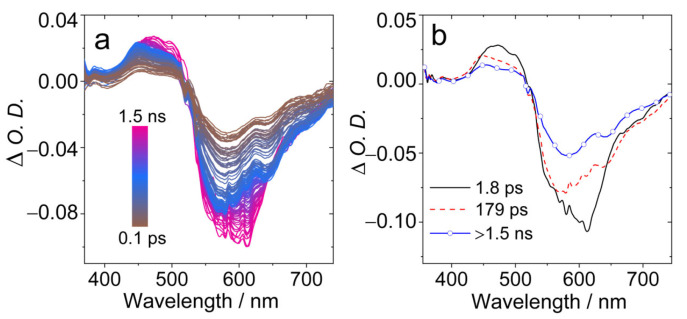
Femtosecond transient absorption spectra of **AN-BDP**. (**a**) Transient absorption spectra at different delay times and (**b**) evolution-associated difference spectra (EADS). EADS were obtained by singular value decomposition (SVD) and global fitting. *λ*_ex_ = 560 nm, *c* = 1.0 × 10^−5^ M in DCM, 20 °C.

In order to study the excited states dynamics, the femtosecond transient absorption (fs-TA) spectra of **AN-BDP** were studied (Figure 7). A broad GSB band centered at ca. 600 nm was observed (Figure 7a), as well as ESA band in the range of 450–520 nm. With global fitting and target analysis, the evolution-associated difference spectra (EADS) were obtained (Figure 7b). The first spectrum has a shoulder negative band at 610 nm, which is assigned to stimulated emission (SE) band; this spectrum is attributed to the Franck–Condon (FC) singlet excited state (S_1_ state). Then within 1.8 ps, the spectrum evolved into the second one, this time is attributed to the vibronic relaxation process. The second spectrum is without significant SE band. Within 179 ps (possibly due to geometry relaxation and solvation changes), the third spectrum was the result, with decays over 1.5 ns. We assign the third spectrum to the relaxed emissive S_1_ state, for which the lifetime is in good agreement with the fluorescence lifetime of **AN-BDP** in DCM (2.3 ns. Figure 3b). No significant formation of the triplet state was observed within the time window of the fs-TA spectrometer (1.5 ns). This is probably due to the slow ISC and low ISC yield of **AN-BDP** (*Φ*_Δ_ = 10%). Previously, a slow ISC was observed for **helical-BDP-1** (ca. 8 ns) [40]; for **helical-BDP-2**, the ISC was determined as 2.57 ns [42].

### 2.6. Time-Resolved Electron Paramagnetic Resonance (TREPR) Spectra

**Figure 8:**
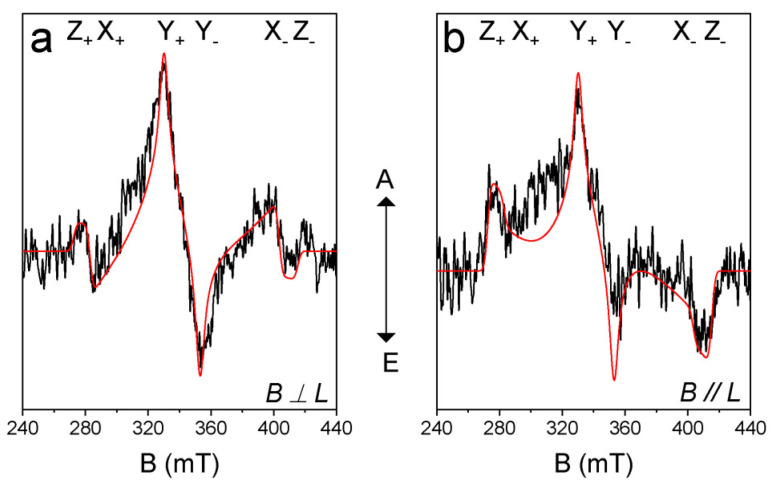
TREPR spectra of **AN-BDP**. The simulations (red lines) and experimental data (black lines) are shown. The polarization of laser and magnetic field is (**a**) perpendicular and (**b**) parallel. Spectra were recorded by excitation of the frozen solution with 580 nm nanosecond-pulsed laser. In TOL/MeTHF (3:1, *v*/*v*). 80 K. Integration time window: 0.0~0.4 μs.

In order to study the electron spin selectivity and the spatial confinement of the triplet-state wave function [43,45,46,58], the TREPR spectrum of **AN-BDP** was recorded (Figure 8). The ESP phase pattern of the triplet TREPR spectrum of **AN-BDP** is (*a*, *e*, *a*, *e*, *a*, *e*); this is different from the ordinary ESP phase pattern of the triplet states of anthracene (*e*, *e*, *e*, *a*, *a*, *a*) and **2I-BDP** (*e*, *e*, *e*, *a*, *a*, *a*) [53] produced by the spin–orbit coupling (SOC) ISC effect. The spin–orbit charge transfer (SOCT) ISC produces a triplet state showing different ESP as compared to that of SOC. The ESP of **AN-BDP** is same to that of **helical-BDP-1** [40], but it is different from that of **helical-BDP-2**, which shows ESP of (*a*, *a*, *e*, *a*, *e*, *e*) [42]. These results indicate the sensitivity of the ESP to the structure of the twisted BDP derivatives.

Simulation of the TREPR spectrum of **AN-BDP** gives the zero-field-splitting (ZFS) *D* and *E* tensors, as well as the population rates of the three sublevels of the triplet state [45,46]. We performed the magneto photoselection (MPS) experiment which allows the determination of the relative orientation of the transition dipole moment (TDM) and ZFS axes. The *D* and *E* values of **AN-BDP** are −71.4 mT and 16.2 mT, respectively (Table 2). The orientation of the TDM (θ, φ) was (30°, 90°), where θ and φ are the polar and azimuthal angles between ZFS tensor and TDM, respectively. From the ZFS parameter *D* and *E* values, we concluded that the electron spin density is *delocalized* on the twisted π-conjugation framework of **AN-BDP**. This is because the magnitude of the *D* and *E* parameters of the triplet state of 2,6-diiodoBODIPY (**2I-BDP**) are −105.0 mT and 23.2 mT, respectively [53], which are larger than that of **AN-BDP**. The magnitude of the *D* parameter is dependent on the dipolar interaction of the electrons (spin–spin coupling, SSC), which is related to the average distance between the two electrons. Spin–orbit coupling (SOC) also contribute to the ZFS *D*. For a non-fused anthracene-BODIPY dyad, the triplet state localized on the BODIPY unit show *D* and *E* parameters of −85.0 mT and 17.5 mT, respectively [53]. We recently reported a twisted BODIPY derivative with larger π-conjugation framework, which shows *D* and *E* parameters of −59.5 mT and 11.0 mT, respectively [40]. These results show that the triplet state of a molecule with larger π-conjugation system gives smaller ZFS *D* value. Interestingly, the *D* and *E* parameters of the triplet state of **AN-BDP** are similar to a recently reported twisted BODIPY derivative, with two peripheral phenyl rings, for which the *D* and *E* parameters are −69.5 mT and 15.5 mT, respectively [42]. The population rates of the three sublevels of the T_1_ state of **AN-BDP** are *P_x_*: *P_y_*: *P_z_* = 0: 1: 0 (Table 2), which are different from the previously reported twisted BDP derivatives [40,42]. Also, the TDM orientation of **AN-BDP** is not coincide with TDM orientation of 2,6-diiodoBODIPY [59].

### 2.7. Theoretical Computation: The Non-Radiative Decay Channel and the ISC of AN-BDP

**Figure 9:**
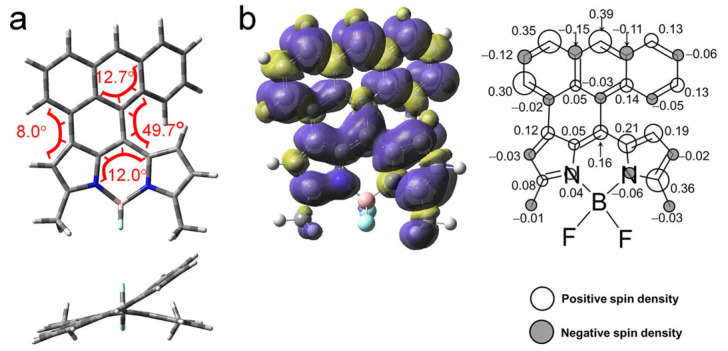
(**a**) Optimized conformations and the dihedral angles of **AN-BDP**. Top, side view; bottom, top view. DFT computation is at UB3LYP/6-31G(d) level with Gaussian 16. (**b**) Isosurfaces of spin density of **AN-BDP** at the optimized triplet state geometries (Isovalue = 0.0004). The color part in (**a**) highlight the torsion of the molecular structure. Calculation was performed at UB3LYP/6-31G(d) level with Gaussian 16. The numbers in the figure indicate the contribution of each atom to the total spin density.

The geometry and the photophysical property of **AN-BDP** were studied in detail with theoretical computation. The optimized ground state geometry indicated a highly twisted molecular structure (Figure 9a). Both the fused anthryl and the BDP units are non-planar. The anthryl unit is twisted by 12.7°, whereas the puckered BDP unit is with torsion of 12°. The dihedral angle between the two units is 49.7°, whereas it is 8.0° on the other side of the fused structure (Figure 9a). These geometry detail was not reported in the previous literature [47], and it is drastically different from the analogue that the anthryl unit is fully fused with the BDP unit, the fully fused analogue takes a planar geometry [54]. The conformation of **AN-BDP** is also different from the recently reported anthryl-BODIPY dyads in which the two units are connected by one single C-C band [52,53,60].

The spin density surface of the T_1_ state of the **AN-BDP** was computed (Figure 9b); the results show that the spin density is fully delocalized on the whole molecular structure, and the distribution on the fused anthryl moiety is significant. This result is in agreement with the smaller ZFS *D* parameter of **AN-BDP** as compared to that of the **2I-BDP**. Note the spin density distribution of **AN-BDP** is also slightly different from that of the **helical-BDP-1**, for which the spin density is mainly confined on the core of the BDP, and the distribution on the peripheral fused phenyl rings are limited [40]. The triplet state spin density distribution is drastically different from the compact orthogonal anthryl-BDP dyads with C-C as linker between the two parts, for which the spin density of the T_1_ state is exclusively confined on the BDP unit [53]. The T_1_ state energy was estimated to be 0.96 eV with TDDFT computation. It is significantly lower than the native BDP (ca. 1.6 eV) and **helical-BDP-2** (ca. 1.5 eV) [42].

**Figure 10:**
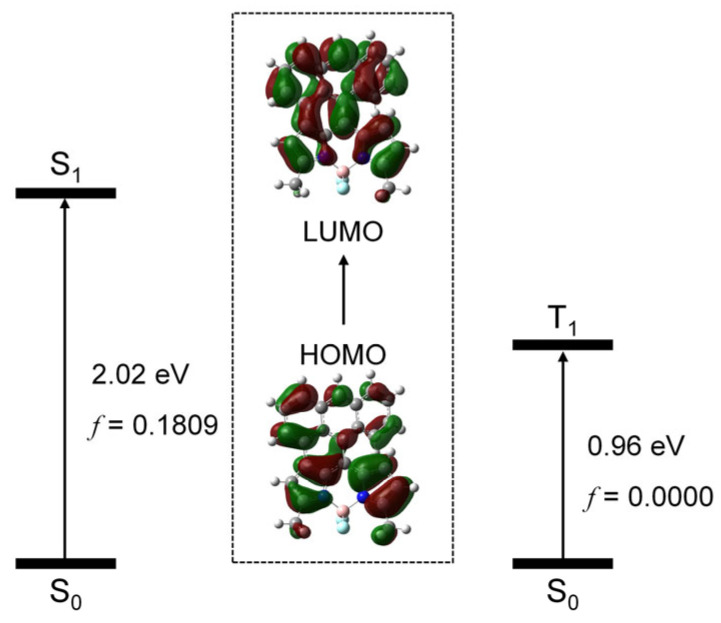
Molecular orbitals involved in the transition from the ground state (S_0_) to the low-lying excited states (S_1_ and T_1_ states). TDDFT computation was performed at B3LYP/6-31G(d) level with Gaussian 16.

We studied the frontier molecular orbitals involved in the transition from ground state (S_0_) to the low-lying electronic excited states (S_1_ and T_1_ states), using the TDDFT method. We found that, for the S_0_ → S_1_ transition, the HOMO and LUMO are involved, and the excitation energy is 2.02 eV, in good agreement with the experimental results (2.14 eV). For the S_0_ → T_1_ transition, the HOMO and LUMO are involved and the excitation energy is 0.96 eV. The HOMO and LUMO are mainly delocalized on the whole molecular structure (Figure 10). Note that, for the transition, no SOC effect was considered; therefore, the oscillator strength for the transition is null.

Using the TDDFT method and the EPR/NMR module of ORCA 6.0 program [61], we carried out a preliminary study of the ISC ability and the triplet state property (mainly the ZFS). Firstly, we checked the spin–orbit coupling matrix elements (SOCMEs) of the S_1_ state to the triplet states, we found that for the S_1_ → T_n_ (n = 1–4), the SOCMEs are negligible (less than 0.5 cm^−1^), indicating that the ISC should be weak for **AN-BDP**. This is in agreement with the experimental results. In order to verify this approach, we also investigate the BODIPY with the same method, which is known for lack of ISC ability [26]. Small SOCMEs with similar magnitude were observed. On the other hand, for the **2I-BDP**, which is known showing efficiency ISC [26], larger SOCMEs were observed: for instance, the SOCMEs are 1.14 cm^−1^, 1.29 cm^−1^, and 460 cm^−1^ for the ISC of S_1_ → T_3_, S_1_ → T_4_, and S_1_ → T_5_, respectively.

Moreover, the ZFS of the triplet state of **AN-BDP** was calculated with the EPR/NMR module of ORCA 6.0 program. For **AN-BDP**, the calculated ZFS *D* is −15.9 mT, which is on the same order of magnitude of the experimental value of −71.7 mT. The contribution of spin–spin coupling (SSC) to the ZFS *D* tensor of **AN-BDP** is −23.5 mT, and the contribution of the spin–orbit coupling (SOC) to the ZFS *D* tensor is 7.6 mT. On the other hand, we studied **2I-BDP** (which is known to have an efficient ISC) with a similar method for comparison. The calculated ZFS *D* is −98.4 mT, which is in good agreement with the TREPR experimental results (−105.7 mT) [53]. In this case, the contribution of SSC to the ZFS *D* tensor is −2.1 mT, and the contribution of SOC to the ZFS *D* tensor is −96.1 mT; this is reasonable because the SOC is strong for **2I-BDP**, due to the heavy atom effect of the iodine atoms in the molecular structure. Furthermore, for the unsubstituted BODIPY, the calculated ZFS *D* is −66.3 mT; again, it is in good agreement with the experimental results (−84.5 mT; note the theoretical computation correctly predicted the trend of the change of the magnitude of the ZFS *D* values) [53]. The contribution of SSC to the ZFS *D* tensor is −73.8 mT, and the contribution of SOC to the ZFS *D* tensor is much smaller (7.4 mT).

For the calculation of the ISC rate constants, the geometries of the states S_1_, T_1_, and T_2_ were optimized by TDDFT using the CAM-B3LYP functional and the def2-SVP basis set. The Hessian matrices of all states were calculated, all stationary points are minimal. For each pair of states, the SOCMEs and their derivatives with respect to all normal coordinates were calculated. The ORCA 6.0 programs [61] were used, in particular the ESD [62] module. These data were subsequently used by our own program [63], which determines the ISC rate constants (both Franck–Condon and Herzberg–Teller terms) as function of the energy gap.

**Figure 11:**
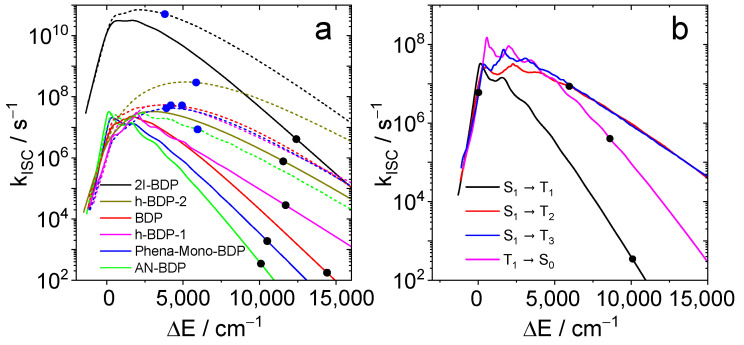
(**a**) Calculated ISC rate constants as function of the energy gap. The colors correspond to **2I-BDP** (black), **BDP** (red), **helical-BDP-1** (magenta) **helical-BDP-2** (brown), **Phena-Mono-BDP** (blue) and **AN-BDP** (green). Full lines are the S_1_-T_1_ rates; dotted lines are S_1_-T_2_ rates. Black dots indicate the calculated S_1_/T_1_ energy gaps; blue dots indicate the calculated S_1_/T_2_ energy gaps. (**b**) ISC rate constants for **AN-BDP** as function of the energy gap for the transitions S_1_-T_1_ (black), S_1_-T_2_ (red), S_1_-T_3_ (blue), and T_1_-S_0_ (magenta). The dots indicate the calculated energy gaps.

The results are summarized in Figure 11. For all compounds, the S_1_ → T_1_ rate constant at the calculated energy gap is smaller than 10^7^ s^−1^. Hence, this channel should not contribute significantly to the decay of S_1_ in these compounds. When T_2_ is considered as final state of the ISC (dotted lines and blue dots), smaller energy gaps and larger rate constants are found. The largest value (5.1 × 10^10^ s^−1^) is obtained for **2I-BDP**. The other rates are still small compared to the radiative rate of fluorescence. Hence, they are compatible with moderate quantum yields for both fluorescence and triplets. The calculated rate constants (3.5 × 10^2^ s^−1^ for T_1_, 8.6 × 10^6^ s^−1^ for T_2_, and 6.0 × 10^6^ s^−1^ for T_3_) are rather small compared to the radiative rate constants. The sum of the latter two comprise about 10% of the radiative rate, which could explain a triplet yield of similar magnitude. The rate constant for the transition T_1_ → S_0_ (4.0 × 10^5^ s^−1^) corresponds to a triplet lifetime of 2.5 µs, whereas the observed lifetime is larger by a factor of ca. 200. This is probably due to the use of the harmonic approximation which leads to overestimation of the rate constant with an increasing energy gap.

**Figure 12:**
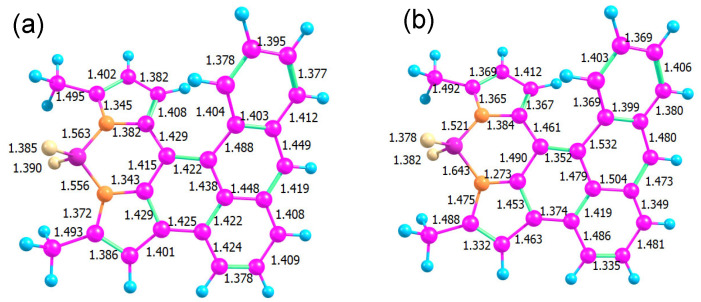
Bond lengths in the optimized structures of the electronic ground state S_0_ (**a**) and the conical intersection (**b**).

The optimized ground state geometry of **AN-BDP** is presented in Figure 12. The low quantum yields of **AN-BDP** both for fluorescence and triplet in polar solvents could be explained by a fast, radiation-less decay of S_1_ directly to the ground state. In a helicene-like structure, a ring closure at the overlapping ends of the helix is a possible path: for example, for a helicene with two thiophene units, such a ring closure was found along a barrierless path from the excited singlet state [63]. The resulting structure is a biradical with almost degenerate singlet and triplet. From this point, another path leads without a barrier back to the molecular singlet ground state. We checked the reaction path for such a ring closure in **AN-BDP** but found a substantial barrier in this case.

Another option is a conical intersection (CI) between S_1_ and S_0_ states at an energy below or near the Franck–Condon point (Figure 12b). Such a conical intersection has been found for **BDP** analogue phenyl–BDP at a geometry where the BDP unit is bent and the phenyl ring is “coplanar” with the BDP unit [55,64,65]. A search for a CI requires a wavefunction of the same quality for S_0_ and S_1_; hence, we cannot use a single reference method (like DFT). We used an algorithm based on the gradient projection method [66] implemented in a home-written program [67] that treats constraints in an orthogonal basis and acts as a frontend to the Firefly quantum chemistry program. Our search for a CI was successful with a surprising result: the main geometrical changes occur in the bond lengths connecting the BDP and the AN units, accompanied by a reorganization of bond alternation in some of the aromatic rings.

**Figure 13:**
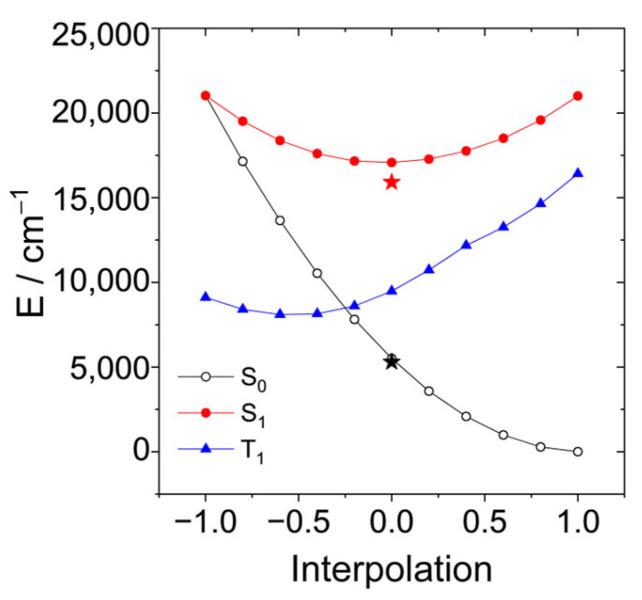
Linear interpolation between the structures of the conical intersection (*r* = −1) and the ground state (*r* = +1). The structures at the endpoints were optimized with state-averaged CASSCF(10|10) and the cc-pVDZ basis. The energy of the triplet state is shown as the blue curve. The optimized energy of the S_1_ state and the corresponding energy of the ground state at this geometry are shown as red and black stars.

A linear interpolation between these structures yields the potential energy curves shown in Figure 13. The optimized energy of the S_1_ state and the corresponding energy of the ground state are shown as red and black stars. The FC-point is at the same energy as in the case when the increasing polarity of the solvent lowers the barrier. Although we do not find a vanishing barrier with this level of theory, the results provide an explanation that the radiation-less process becomes more efficient in more polar solvents. (Note that the transition from the S_1_ to the S_0_ state usually occurs when both states approach within the energy of a few vibrational quanta.) This can explain the absence of fluorescence and the missing triplet yield of **AN-BDP**.

In the CI, there is no barrier between both structures on the surfaces of S_0_ (black), S_1_ (red), and T_1_ (blue). Therefore, decay to the CI can compete with vibrational relaxation. The optimized structure of the S_1_ state is somewhat below the CI. Hence, when vibrational relaxation occurs before the system can reach the CI, internal conversion via the CI requires an activation energy. Table 3 shows these activation energies calculated at the same level of theory, CASSCF(10|10)/cc-pVDZ, but considering various solvents with the PCM model.

## 3. Materials and Methods

### 3.1. Analytical Measurements

All chemicals used in the synthesis are analytically pure and were used as-received. Solvents were dried and distilled before used for synthesis. The UV–vis absorption spectra were recorded on an Agilent 8453 UV–vis spectrophotometer (Agilent Ltd., Foster City, CA, USA). Fluorescence spectra were recorded on an RF-5301 PC spectrofluorometer (Shimadzu Ltd., Kyoto, Japan). Luminescence lifetimes were measured on an OB920 fluorescence lifetime instrument (Edinburgh Instruments Ltd., Edinburgh, UK).

### 3.2. Nanosecond Transient Absorption Spectra

The nanosecond transient absorption spectra were measured on an LP980 laser flash photolysis spectrometer (Edinburgh Instruments Ltd., UK). The samples were photoexcited with an OpoletteTM 355II+UV nanosecond-pulsed laser (OPOTEK, Carlsbad, CA, USA; typical pulse length: 7 ns. Pulse repetition: 20 Hz. Peak OPO energy: 5 mJ). The wavelength is tunable in the range 410–2200 nm. Lifetime values were obtained with the L900 software (https://www.edinst.com/product/l900-software/, accessed on 26 January 2026) by fitting exponential functions to the decay traces. The intrinsic triplet state lifetimes were obtained with a kinetic model that the TTA self-quenching effect is considered [53,55]. All samples in flash photolysis experiments were deaerated with N_2_ for ca. 15 min before measurement.

### 3.3. TREPR Spectroscopy

The magnetic resonance experiments were carried out in frozen solutions of the compounds with a mixture of toluene/MeTHF (3:1, *v*/*v*) as solvent. Optical excitation was carried out with an optical parametric oscillator (OPO) system (LP603, SolarLS, Minsk, Belarus) pumped by an Nd:YAG laser (LQ 629 SolarLS, Minsk, Belarus) with a pulse energy of 1 mJ with pulse length = 10 ns. The repetition rate of the laser was set to 100 Hz. Polarization for the MPS experiments was obtained using a Glan laser polarizer (SolarLS, Minsk, Belarus) in combination with a half-wave plate. The TR CW EPR spectra were obtained by the summation of the data in different time windows after the laser pulse. The EPR spectra were simulated using the EasySpin (6.0.4) package implemented in the MATLAB (R2021b) programming language [68].

### 3.4. Theoretical Computation

The ground states geometry of the compound was optimized with Gaussian 16 [69]. The TDDFT computation was done at the optimized ground state geometry. The excited state property including the ISC rate constants were calculated with ORCA 6.0 program [61]; this involves optimization of the geometries of the initial and final states of each transition, including calculation of the hessian matrices. The CAM-B3LYP functional and the def2-SVP basis set were used throughout, using DFT for the ground state and TDDFT for all excited states. The search and optimization of the conical intersection used the CASSCF method with the cc-pVDZ basis set using the Firefly QC package [70] (which is partially based on the GAMESS (US) [71] source code) with our own optimizer [67] as frontend.

## 4. Conclusions

In summary, we studied the intersystem crossing (ISC) of an anthracene-fused boron–dipyrromethene (**AN-BDP**) derivative, which has highly twisted molecular structure. The fused anthryl and the BDP in **AN-BDP** units both adopt distorted geometry (with ca. 12° of torsion), and there is large dihedral angle between the two units (ca. 49.7°). The fluorescence quantum yields are highly dependent on the solvent polarity (59~3% in toluene and acetonitrile); yet, the fluorescence emission wavelength does not change in different solvents. The nanosecond transient absorption spectra indicate that the triplet state is long-lived, with intrinsic triplet state lifetime of 551 μs. Interestingly, the severely twisted structure only show a moderate ISC yield (10%), which is different from the trend found for helicene. Femtosecond transient absorption spectra indicate the intersystem crossing (ISC) is slow (>1.5 ns). The time-resolved electron paramagnetic resonance (TREPR) spectra show smaller zero-field-splitting *D* (−71.4 mT) and *E* tensors (16.2 mT) as compared to the triplet excited state of the iodinated native BDP (−104.6 mT and 22.8 mT, respectively), inferring that triplet-state wave function of the compound is delocalized over the twisted molecular framework. Theoretical computation indicated a decreased energy barrier from the relaxed S_1_ state to a conical interaction (CI) of the S_1_ and the S_0_ state potential curves in polar solvents, which agrees with the quenched fluorescence in polar solvents compared to that in non-polar solvents.

## Figures and Tables

**Table 1:** The photophysical properties of **AN-BDP**.

	Solvent	*λ*_abs_ ^1^ (*ε* ^2^)	*λ*_F_ ^3^	*τ*_F_ ^4^	*Φ*_F_ ^5^	*Φ*_Δ_ ^6^	*τ*_T_ ^7^	*k_r_* ^8^	*k_nr_* ^9^
**AN-BDP**	HEX	580 (4.50)	637	3.5	0.62	0.05	432.1	1.77	1.09
	TOL	590 (4.20)	645	3.1	0.59	0.06	662.4	1.90	1.32
	DCM	583 (4.42)	646	2.3	0.32	0.10	551.0	1.39	2.96
	ACN	582 (4.40)	642	0.4	0.03	0.05	472.4	0.75	24.3

^1^ Absorption wavelength, in nm, *c* = 1.0 × 10^−5^ M. ^2^ Molar absorption coefficient. *ε* values are in 10^4^ M^−1^ cm^−1^. ^3^ Fluorescence emission maxima wavelength, in nm. ^4^ Fluorescence lifetimes, in ns, *λ*_ex_ = 510 nm, *c* = 1.0 × 10^−5^ M. ^5^ Absolute fluorescence quantum yield. ^6^ Singlet oxygen quantum yield. Methylene blue (MB) as standard (*Φ*_∆_ = 0.57 in DCM). ^7^ Intrinsic triplet state lifetime, in μs. Obtained by fitting based on the triplet–triplet–annihilation self-quenching effect considered. ^8^ Radiative transition rate constant, *k*_r_ = *Φ*/*τ*_F_, in 10^8^ s^−1^. ^9^ Non-radiative transition rate constant, *k*_nr_ = (1 − *Φ*)/*τ*_F_, in 10^8^ s^−1^.

**Table 2:** ZFS parameters (*D* and *E*) and relative population rates *P_x,y,z_* of the zero-field spin states of compounds. The compounds used in the study as reference are also presented.

Compound	*D* (mT)	*E* (mT)	*P_x_*:*P_y_*:*P_z_*^ 1^
**AN-BDP**	−71.4	16.2	0:1:0
**2I-BDP** ^ 2^	−104.6	22.8	0:0.13:0.87
**helical-BDP-1** ^ 2^	−59.5	−11.0	0.83:0:0.17
**anthracene** ^ 2^	−77.8	−9.0	0.4:0.6:0

^1^ Representative population of the zero-field spin states normalized to 1. ^2^ Literature results.

**Table 3:** Energy barrier (Δ*E*, in cm^−1^ units) from the optimized structure of S_1_ towards the CI in various solvents (ACE: acetone; TOL: toluene; CHX: cyclohexane).

Solvent	H_2_O	CH_3_NO_2_	EtOH	ACE	DCM	THF	CHCl_3_	DMSO	CH_3_OH	CCl_4_	TOL	CHX
Δ*E*/cm^−1^	3204	3258	3315	3344	3566	3629	3838	4184	4202	4379	4728	4795

## Data Availability

The original contributions presented in this study are included in the article. Further inquiries can be directed to the corresponding author(s).
